# Cost-Analysis of the Different Treatment Modalities in X-Linked Dystonia–Parkinsonism

**DOI:** 10.3389/fneur.2019.00500

**Published:** 2019-05-08

**Authors:** Ranhel C. De Roxas, Roland Dominic G. Jamora

**Affiliations:** ^1^Department of Neurosciences, Philippine General Hospital, University of the Philippines Manila, Manila, Philippines; ^2^Department of Neurosciences, College of Medicine – Philippine General Hospital, University of the Philippines Manila, Manila, Philippines; ^3^Movement Disorders Service, Institute for Neurosciences, St. Luke's Medical Center, Quezon City, Philippines

**Keywords:** cost analysis, dystonia, parkinsonism, XDP, healthcare cost, lubag

## Abstract

**Background:** X-linked dystonia-parkinsonism (XDP) is a debilitating disease endemic in the Philippines. Several oral medications as well as botulinum toxin A (BoNT-A) injection and deep brain stimulation (DBS) surgery appear to be the cornerstone of treatment in XDP, which are commonly used in combination. Being a chronic progressive disease, it is an economic burden to the patient and their families. Thus, we aim to perform a comparative analysis of the associated healthcare costs for the therapeutic options used in XDP.

**Methodology:** A questionnaire assessing the healthcare costs in the management of XDP was designed and administered through an interview with the XDP patients or their caregivers. We analyzed the data and a bootstrap analysis was also done to obtain a more generalizable estimation of the costs.

**Results:** A total of 110 gene-positive XDP patients were included in this study. The mean total annual cost per patient was USD 4,861.23 (USD:PHP 1:50, as of January 8, 2018). More than half of the patients (*n* = 61, 55.5%) received both oral medications and BoNT-A injection while 42 patients (38.2%) received oral medications alone. Only seven patients underwent DBS with a reported estimated cost of USD 50,931.43. The bootstrap analysis confirmed the estimates done in this study.

**Conclusion:** The estimated costs in the management of XDP was shown to be 30 times the average annual health expenditure of an average Filipino. This calls for more government effort to provide comprehensive care for chronic and debilitating diseases such as XDP.

## Introduction

X-linked dystonia-parkinsonism (XDP, *Lubag*, OMIM 314250) is an adult-onset, predominantly male, inherited, debilitating and progressive disease endemic in the Philippines manifesting as dystonia and parkinsonism, usually occurring during the third and fourth decade of life (1). Majority of patients initially present with focal dystonia becoming generalized, eventually progressing to parkinsonism presenting as tremors, bradykinesia and gait instability within 5 years of onset (1). Due to its presentation, patients have decreased quality of life and shortened life span (2). Its diagnosis is a burden not only to the patient but also to the family because of its stigma and genetic nature.

Oral medications such as anti-cholinergics, anti-convulsants, anti-histamines, sedatives, and even carbidopa/levodopa have been tried, but these drugs have shown inconsistent to no benefit among patients (3, 4). Substantial improvements were also found in patients given with botulinum toxin A (BoNT-A) injections especially those who were presenting with oromandibular, lingual, and truncal dystonia (5). Surgery, in the form of deep brain stimulation (DBS) is also an option in the treatment of XDP. Bilateral pallidal DBS have shown significant long term improvement in the dystonia of the patients (6–8). However, 23% of XDP patients remain to be either wheelchair-borne or bed-bound (1).

The Philippines has a population of 100.98 million as of August 2015 (9). Philippine healthcare is still an out-of-pocket basis which account for 56% of the total health expenditure of the country (10). Although the Philippine Health Insurance Corporation (PhilHealth) is improving on its benefit schemes for a defined set of services for a predetermined rate and is currently making great efforts to subsidize the needs of the Filipinos, there are still a number of diseases such as XDP, which are not yet covered. This makes access to healthcare difficult, more so to neurological diseases in which resources and manpower are also scarce (10, 11). On the average, Filipinos only spend approximately USD 160.00 for health expenditures annually (11). However, for an XDP patient, this does not suffice. Because of the high economic burden of the disease to the patient as well as to the family, many of them resort to looking for financial assistance from government and non-government institutions and charitable foundations. At present, there is still no data on the average health consumption of an XDP patient, which could help healthcare providers in giving advice to the patient and the family and could also guide the PhilHealth in providing assistance to the patients. Thus, this study aims to address the gap in comparing the economic data on the treatment options and perform a comparative analysis of the associated healthcare costs using oral medications, BoNT-A injection and DBS among patients with XDP.

## Materials and Methods

We consecutively enrolled XDP patients seen at the XDP Clinics at the Philippine General Hospital in Manila and in Health Centrum, Roxas City.

A questionnaire was designed by the authors based on their experiences on the needs of the XDP patients seen at the clinics. It was administered through an interview with the patient or their caregivers. The questionnaire focused on the total healthcare costs, defined as services, intervention, medications, and miscellaneous costs associated with the treatment of XDP over a period of 5 years. The inpatient costs, if any, were also recorded. Because of the high variability in the treatment, the questionnaire included all the possible combinations. A microcosting approach was utilized.

The analyses were performed using STATA SE version 13 (StataCorp, Texas, USA). In order to obtain a more generalizable estimation of the costs, a bootstrap analysis was performed using the STATA program. We used a sample size of 50 and a re-sample of 1,000, calculating the main statistics expressed as mean (median; 2.5 percentile; 97.5 percentile). A comparison of costs between the different treatment was also analyzed using the non-parametric Kruskal-Wallis one-way analysis of variance test. The level of significance was set at 5%.

This study was carried out in accordance with the recommendations of University of the Philippines Manila Research Ethics Board (UPMREB). The protocol was approved by the UPMREB (2017-109-01). All subjects gave written informed consent in accordance with the Declaration of Helsinki.

## Results

A total of 110 gene-positive XDP patients were included in this study. Majority (*n* = 82, 75%) of the patients were from the XDP Clinic in Roxas City. The mean age of the patients was 48.7 ± 8.22 years (range, 32–75 years). Majority of the patients (*n* = 88, 80%) had their onset on symptoms during their third to fourth decade of life with the mean age of onset at 42 ± 7.08 years. The majority of the XDP patients (*n* = 88, 80%) were married (80.0%) and unemployed (*n* = 101, 92%). Only 44 patients (40%) were able to finish tertiary education.

The mean total annual cost per patient was USD 4,861.23 ± 13,070.89 (1 USD = 50 PHP, as of January 2018) and the median cost was USD 1,969.02. The biggest expenditure of an XDP patient was the DBS (USD 50,391.43), followed by tube gastrostomy (USD 2,200) and BoNT-A injections (USD 1,528.48 per annum; see Table 1). There were 7 patients who underwent DBS and the cost reported included the surgery, imaging, neuropsychiatric evaluation, and transportation. It should be noted that ~90% of the expenses for the DBS surgery were incurred in the first year of treatment and all of these patients received external funding for the DBS. Two patients had replacement of the DBS battery, costing USD 30,000.00. Two other patients had tube gastrostomy due to progressive dysphagia. There were 66 (60%) patients who reported receiving BoNT-A injections every 3 months. None of our patients was placed in a nursing home.

**Table 1:** Unit cost of the healthcare resources identified in the treatment of XDP^*^.

**Breakdown of costs**	**Average unit cost/annum (in USD)**
Specialist visit	101.25
Transportation	72.61
Blood tests	98.77
Imaging	177.39
Genetic testing	100.00
Hospitalization	355.65
Pharmacologic treatment	445.75
Botulinum toxin A injection	1,528.48
Deep brain stimulation	50,391.43
Others (PEG insertion)	2,200.00
Nursing homes	–

* *USD 1.00, PHP 50.00, as of January 8, 2018*.

All patients were noted to be taking at least one oral medication for the symptom control of XDP. The most commonly used medication was biperiden, costing an average of USD 12.15 per month (see Table 2). This was followed by clonazepam (USD 7.32 per month) and carbidopa/levodopa (USD 28.31 per month). However, the most expensive medication was zolpidem (USD 59.54 per month).

**Table 2:** Average costs of the oral medications used in XDP patients^*^.

**Oral medications**	**Percentage**	**Average cost/month (USD)**
Biperiden	90.9	12.15
Clonazepam	76.4	7.32
Carbidopa/levodopa	35.5	28.31
Vitamin B complex	10.9	8.09
Zolpidem	7.3	59.54
Diphenhydramine	2.7	8.40
Escitalopram	2.7	32.40
Baclofen	1.8	38.70
Vitamin C	1.8	4.80
Amitriptyline	0.9	12.60
Vitamin E	0.9	14.40
Paracetamol+Tramadol	0.9	21.00
Trihexyphenidyl	0.9	22.50

* *USD 1.00, PHP50.00, as of January 8, 2018*.

More than half of the patients (*n* = 61, 55.5%) received both oral medications and BoNT-A injections while 42 patients (38.2%) received oral medications alone. A small percentage (*n* = 5, 4.5%) of the XDP patients received oral medications, BoNT-A injection and DBS. Table 3 shows the annual average cost per patient and the total cost for 5 years using the different options in the treatment of XDP. There was no significant difference between the use of oral medications only and oral medications with BoNT A injections. However, there was a significant difference with the use of DBS with the other treatment modalities (*p* value ≤ 0.001)

**Table 3:** Comparison of the average cost of the different treatment options in XDP^*^.

	**N (%)**	**Average cost/year (in USD)**	**Total cost for 5 years (in USD)**	***p*-value**
Oral medications only	42 (38.2)	445.75 ± 3, 545.94	2,228.73	-
BoNT-A injection only	0 (0.0)			
DBS only	0 (0.0)			
Oral medications + BoNT-A injection	61 (55.5)	*1, 562.03*±*785.47*	7,810.19	0.625
Oral medications + DBS	2 (1.8)	*70, 764.53*±*9, 530.01*	72,547.52	<0.001
Oral medications + BoNT-A injection + DBS	5 (4.5)	*43, 823.72*±*9, 173.65*	45,606.72	<0.001

* *USD 1.00, PHP50.00, as of January 8, 2018*.

More than half of the patients (*n* = 57, 51.8%) reported moderate improvement of the symptoms on treatment (oral medications only = 16; oral medications + BoNT-A injection = 34; oral medications + DBS = 2; oral medications + BoNT-A injection + DBS = 5). This means that they experience more than 50% reduction on the severity of symptoms. On the other hand, 48.2% (*n* = 53) of the patients reported only minimal improvement of <50% in terms of symptom control (oral medications only = 26; oral medications + BoNT-A injection = 27). No patient reported complete resolution of symptoms.

A bootstrap analysis was done, and it confirmed similar results with the different healthcare resources identified in the treatment of XDP.

## Discussion

This is the only study which tried to estimate the costs related to the management of XDP. As shown, the patients spent a considerable amount of money prior to the diagnosis of XDP because of multiple physician visits. This delay in the diagnosis may be explained because of the high variability of the clinical presentation of the disease and probably, some clinicians are not adept in diagnosing XDP (1).

This study also demonstrated that the current management of XDP necessitates multiple approaches. The use of an anticholinergic and benzodiazepine remains to be the mainstay in pharmacologic treatment. However, it should be noted that in more severe and generalized dystonia, polypharmacy may not have any effect anymore and zolpidem may be the only potentially effective agent in such cases (3). The use of carbidopa/levodopa remains widespread, despite a recent paper saying it is ineffective for XDP (4). The use of BoNT-A injection as an adjuvant symptomatic treatment was shown to have no significant difference in the use of oral medications alone. Only a small number of patients in this study underwent DBS, which is considered as the best option in advanced XDP that is refractory to other forms of treatment (6–8). In the Philippines, only two institutions are equipped in performing the surgery and it is not covered by PhilHealth (6). As shown here, DBS is a very expensive option for XDP patients and all patients who received it in this study were part of a study for XDP and were fortunate to receive funding for the surgery (7). Moreover, it is not only expensive initially but requires frequent visits to the physician for programming and eventually a replacement of the DBS battery, which further adds to the cost.

On the average, an XDP patient need to spend USD 4,861.23 annually for the management of their symptoms despite the incomplete resolution. This amount is significantly lower compared to other more common movement disorders in the region such as Parkinson's disease where the annual treatment cost is estimated to be USD 10,129.00 (12). However, the average minimum daily wage in the Philippines (depending on the industry and the region) is only USD 9.39–10.17/day (10). The average Filipino annual family income is USD 3808.72, while the average annual family expenditure is USD 3131.62 (10).

However, this amount (USD 4,861.23) is ~30 times the allotted annual health expenditure of an average Filipino (USD 160.00) and more than 50% of which is considered out-of-pocket expenditure (13). A patient will need about USD 34,028.61 for the next 7 years (diagnosis at age 48, death at 55 years) granting that his medications remain the same, which is unlikely. The mean age at death of XDP patients was 55.59 years (1). Our patients are spending less than the reported cost in this paper due to the help extended by various private foundations (Sunshine Care Foundation, Let's Save the Brain Foundation) as well as government institutions such as the Philippine Charity Sweepstakes Office which provides selected oral medications and BoNT-A vials for free, though not on a regular basis.

There are certain limitations to this study. First, the study is not a cost effectiveness analyses as there is no available comparative outcome data in XDP. Moreover, as this was an interview using a questionnaire, a recollection bias may have occurred. Also, this study did not look on the disability-adjusted life years or the income loss of the XDP patients since the goal of this study was to be a basis on the treatment decisions of supporting organizations, government institutions, and physicians for these patients. Nevertheless, this was the only study that showed the expenses incurred by a patient with XDP.

## Conclusion

The cost of managing XDP was shown to be 30 times the average annual health expenditure of an average Filipino. This calls for more government effort to disaggregate the poor distribution of healthcare resources and to provide comprehensive care for chronic and debilitating diseases such as XDP.

## Data Availability

The raw data supporting the conclusions of this manuscript will be made available by the authors, without undue reservation, to any qualified researcher.

## Ethics Statement

This study was carried out in accordance with the recommendations of University of the Philippines Manila Research Ethics Board (UPMREB). The protocol was approved by the UPMREB (2017-109-01). All subjects gave written informed consent in accordance with the Declaration of Helsinki.

## Author Contributions

RD: study concept and design, acquisition of data, analysis and interpretation, writing of the initial draft, and critical revision of manuscript. RJ: study concept and design, analysis and interpretation, critical revision of manuscript for intellectual content, and study supervision.

### Conflict of Interest Statement

RJ serves on the advisory board of Lundbeck Phils and Torrent Phils. He has received honoraria from the Philippine offices of Allergan, Innogen, Lundbeck, Medichem, Natrapharm, Sandoz, Sun, Torrent, and Zydus. He has research grants from the Collaborative Center for X-linked Dystonia Parkinsonism and the Philippine Neurological Association. The remaining author declares that the research was conducted in the absence of any commercial or financial relationships that could be construed as a potential conflict of interest.
